# Automated Auricular Surface Temperature Monitoring in Asian Elephants Using Deep Learning and Infrared Thermography

**DOI:** 10.3390/ani16182870

**Published:** 2026-09-11

**Authors:** Ziluo Chen, Yaya Zhao, Mingwei Bao, Fangyi Zhou, Qingzhong Shen, Xianming Guo, Li Zhang

**Affiliations:** 1 Key Laboratory of Biodiversity Science and Ecological Engineering, Ministry of Education, Beijing Normal University, Beijing 100875, China; 202421200065@mail.bnu.edu.cn (Z.C.); zhaoyy6898@163.com (Y.Z.); 2 Xishuangbanna Wild Elephant Valley Asian Elephant Rescue and Breeding Center, Jinghong 666106, China; baomingwei-11@163.com (M.B.); zhoufangyi@mail.bnu.edu.cn (F.Z.); 3 Xishuangbanna National Nature Reserve, Jinghong 666106, China; sqz1196@163.com (Q.S.); 2138519g@163.com (X.G.)

**Keywords:** Asian elephant, infrared thermography, auricular temperature, thermal window, animal welfare, deep learning, YOLO, non-invasive monitoring

## Abstract

Asian elephants (*Elephas maximus*) face substantial thermoregulatory constraints because of their large body size, low relative surface area, sparse hair, and lack of functional sweat glands. Reliable body temperature measurement is essential for assessing thermal status and evaluating welfare in both wild and managed populations, but conventional rectal thermometry requires close physical contact, animal training, and repeated manual handling, making high-frequency, continuous, large-scale monitoring impractical. This study developed a non-invasive framework for automatically detecting the outer ear and extracting auricular surface temperature from infrared thermograms. Rectal temperature, regional surface temperatures, ambient temperature, and relative humidity were measured synchronously in eight semi-captive Asian elephants, yielding 425 matched observations. The associations between rectal temperature and the surface temperatures of three anatomical regions (head, outer ear, torso and limbs) were analyzed using repeated-measures correlation accounting for the non-independence of repeated measurements. Mean outer-ear temperature showed the strongest within-individual association with rectal temperature (*r_rm_* = 0.395, *p* < 0.001), identifying the outer ear as the optimal thermal window for subsequent automated monitoring. Eight lightweight YOLO models—YOLOv5n, YOLOv5s, YOLOv8n, YOLOv8s, YOLO11n, YOLO11s, YOLO26n, and YOLO26s—were trained on 2178 annotated infrared images and evaluated on an independent 194-image test set from extra elephants. Model performance was assessed using detection metrics, inference speed, Bland–Altman agreement, Taylor diagram statistics, and a weighted multi-criteria score with Monte Carlo sensitivity analysis. YOLO11n achieved the best overall performance, with an mAP50 of 0.933 and an inference speed of 164 frames per second. The proposed framework provides an efficient method for automated auricular temperature monitoring and has potential applications in elephant welfare management and remote physiological surveillance.

## Simple Summary

Asian elephants have difficulty dissipating body heat because of their large body size, low surface-area-to-volume ratio, and almost complete absence of sweat glands. Measuring internal body temperature is important for evaluating their thermal status, but conventional methods require close contact, animal cooperation, and repeated manual handling. This study examined whether infrared thermal images could be combined with deep learning to monitor elephant ear temperature automatically. Surface temperature, rectal temperature, and environmental conditions were measured simultaneously in eight semi-captive Asian elephants. The outer ear was identified as the body region most closely associated with rectal temperature. Eight lightweight object-detection models were then trained and compared using 2372 infrared images. All models detected the outer ear effectively, and YOLO11n provided the best overall balance between temperature-extraction performance and processing speed. The approach enables rapid, non-invasive, and repeatable monitoring of auricular surface temperature. With further validation under different environmental and health conditions, it may support routine welfare assessment and early identification of thermal abnormalities in managed elephants and may contribute to remote physiological monitoring of wild elephants.

## 1. Introduction

The Asian elephant (*Elephas maximus*) is a large-bodied homeothermic mammal that must continuously regulate heat exchange through behavioral and physiological mechanisms to maintain thermal balance. However, its small surface-area-to-volume ratio, sparse body hair, and near absence of sweat glands limit its capacity to dissipate heat through the skin, raising concerns regarding its ability to cope with changing thermal environments [1]. Asian elephants primarily inhabit tropical and subtropical regions where ambient temperatures may approach or exceed body temperature. Under climate change, many of their original habitats are expected to become warmer and drier [2], while extreme heat and cold events may become more frequent. These changes may threaten both the survival of wild Asian elephants and the welfare of elephants maintained under human care.

Understanding the thermal adaptability of Asian elephants requires reliable measurement of core body temperature. Previous research reported a mean core temperature of approximately 36.1 °C in Asian elephants, with a recorded range of 35.9–37.1 °C and modest variation in response to ambient temperature [3]. Conventionally, elephant core temperature is approximated by manually measuring rectal temperature. This method requires animals to be trained to cooperate while conscious, involves close contact and repeated handling, and is unsuitable for collecting large amounts of high-frequency data.

Automated approaches to monitoring core body temperature have consequently received increasing attention. These include implantable microchips and biosensors [4,5], as well as temperature sensors placed at peripheral or internal anatomical sites [6]. Several small, waterproof, and corrosion-resistant temperature loggers have been used to measure the core temperature of Asian and African elephants at predetermined intervals [7]. Kinahan et al. trained captive African savanna elephants to swallow iButton temperature loggers and recovered the devices after they passed through the digestive tract, thereby documenting daily rhythms in core body temperature [8]. Weissenböck et al. developed reusable biotelemetric capsules that recorded gastrointestinal temperature and transmitted measurements remotely in both Asian and African elephants [3]. Their results indicated that Asian elephant core temperature followed a circadian rhythm, reaching its highest level around midday and its lowest level in the early morning, broadly corresponding to daily changes in ambient temperature. Nevertheless, orally administered temperature sensors have practical limitations, including damage caused by chewing, uncertain gastrointestinal transit times, and loss of devices after defecation. A more efficient and less invasive method for monitoring elephant thermal status is therefore needed.

Surface temperature provides important information about heat dissipation and thermoregulation in elephants [9]. However, unlike core temperature, body-surface temperature is directly exposed to the environment and varies considerably among anatomical regions. Measurements obtained from a single location may therefore not represent the thermal state of the entire animal. Infrared thermography (IRT) is a non-invasive technique that visualizes spatial patterns of surface temperature and has increasingly been applied to the physiological monitoring of mammals [10]. Its advantages include remote operation, rapid data acquisition, minimal disturbance to animals, and the ability to characterize temperature distributions over relatively large body regions. Consequently, IRT has been used in physiological and ecological studies for disease detection, reproductive monitoring, behavioral analysis, and assessment of animal thermal status [11,12]. The accuracy of IRT is nevertheless affected by environmental, individual, and technical factors. Environmental influences include direct solar radiation, rainfall, wind, ambient temperature, and relative humidity. Technical factors include the distance between the animal and the camera and the angle or field of view. Individual characteristics, including hair density, hair color, coat condition, skin thickness, and surface moisture, may further influence the quantification of thermal changes [11,12]. Large mammals with sparse hair and largely exposed skin, including elephants and rhinoceroses, are therefore particularly suitable subjects for infrared thermographic studies [13].

Infrared thermography has previously been used to investigate the physiological ecology and thermoregulation of elephants. Previous studies have consistently demonstrated that the auricle acts as a major thermal window due to its extensive vascular network and large exposed surface area, with pronounced temperature responses observed during physical activity and cold exposure [14,15,16]. IRT has also shown clinical value for early detection of auricular inflammatory lesions, as localized hyperthermia precedes visible clinical signs [17]. Overall, the external ear is a functionally critical site for adaptive thermoregulation in Asian elephants, with potential application value in early health monitoring.

Although IRT is suitable for measuring the external-ear temperature of Asian elephants, defining a region of interest and extracting temperature values manually from individual thermograms is time-consuming and delays real-time monitoring [18]. Computer-vision methods based on artificial intelligence have therefore become increasingly important in automated animal monitoring. Deep-learning object-detection algorithms can locate animals or anatomical regions accurately even in complex backgrounds or when targets are partially obscured, thereby reducing the need for repeated manual image processing [19]. The You Only Look Once (YOLO) family comprises single-stage object-detection models characterized by fast inference, and optimized YOLO architectures have been shown to achieve robust recognition performance in complex scenes, making them well suited for real-time applications [20,21]. These properties have led to widespread adoption of YOLO models for animal detection, classification, and individual monitoring in wildlife studies.

When integrated with infrared thermography, deep-learning pipelines have been developed primarily for poultry and livestock, including pigs, dairy cows, and sheep [22,23,24,25]. These pipelines can be broadly grouped into three technical directions: anatomical region detection paired with direct temperature extraction [26], pixel-level segmentation for refined regional temperature measurement [27], and machine-learning models that infer internal temperature or thermal status from surface thermal patterns [28,29,30]. Substantially, detection accuracy alone is insufficient to validate an automated thermal-monitoring system, the agreement between automatically and manually extracted temperatures must be rigorously evaluated [24,26]. Equally important is the a priori selection of an appropriate anatomical region. Target regions must be validated for physiological relevance to core body temperature, rather than being selected solely for technical convenience [31].

Collectively, these studies demonstrate the technical feasibility of combining anatomically appropriate thermal regions with computer vision for non-invasive physiological monitoring. Nevertheless, two critical knowledge gaps remain. First, most automated thermography studies directly select anatomical targets without prior systematic validation of their physiological association with core body temperature, risking that the monitored metric lacks clear biological interpretability. Second, no such automated thermal monitoring framework has been developed for Asian elephants—a species with unique thermoregulatory constraints, whose auricle is not only physiologically central to heat exchange, but also technically suitable for object detection due to its distinct shape and high thermal contrast.

To address these knowledge gaps, the objectives of this study were therefore to: (1) compare the relationships between rectal temperature and the surface temperatures of three anatomical regions—the head, outer ear, and torso-and-limb region—to identify the most informative region for automated monitoring; (2) train and compare eight lightweight YOLO models for automatic detection of the selected region in infrared images; (3) extract mean and maximum temperatures from the detected region by mapping model-generated bounding boxes to the original temperature matrices; and (4) evaluate and rank the models using detection performance, inference speed, and agreement between automatically and manually extracted temperatures. The resulting framework was intended to provide a rapid and non-invasive method for monitoring auricular surface temperature in Asian elephants, with potential applications in routine welfare assessment, thermal-status surveillance, and future remote monitoring of wild individuals. In addition, this work delivers four key novel contributions: it is the first to systematically compare three candidate anatomical thermal windows in Asian elephants using repeated-measures correlation accounting for repeated within-individual measurements; it develops the first deep-learning-based automated pipeline for outer-ear detection and temperature extraction in Asian elephant infrared thermograms, evaluating eight lightweight YOLO architectures across four generations; it proposes a multi-criteria model-selection framework integrating conventional detection metrics, temperature-agreement metrics and practical deployment considerations, with robustness verified through Monte Carlo sensitivity analysis; and it demonstrates that the nano-scale YOLO11n achieves a favorable balance of temperature-extraction accuracy and inference speed, supporting potential edge-deployment for real-time, non-invasive thermal monitoring in managed elephant facilities.

## 2. Materials and Methods

### 2.1. Study Site and Animals

This study was conducted from August 2025 to June 2026 at the Wild Elephant Valley Scenic Area in Mengyang Town, Jinghong City, Xishuangbanna Dai Autonomous Prefecture, Yunnan Province, China (22°10′ N, 100°51′ E; elevation: 795 m above sea level). The study subjects were Asian elephants housed at the Yunnan Asian Elephant Rescue and Breeding Center. Eight semi-captive Asian elephants were included in the study, comprising five females and three males. The elephants ranged from 3 to around 40 years of age (Table S1). All elephants were habituated to routine contact with animal-care personnel.

### 2.2. Data Collection

#### 2.2.1. Data-Collection Equipment

Rectal temperature, infrared thermograms of the body surface, ambient temperature, and relative humidity were recorded using an iButton DS1922L temperature logger (Maxim Integrated Products, Inc., San Jose, CA, USA), a Fluke TiS55+ infrared thermal camera (Fluke Corporation, Everett, WA, USA), and a BENETECH GM1365 temperature and humidity data logger (Shenzhen Benetech Instrument Co., Ltd., Shenzhen, Guangdong, China), respectively. The iButton DS1922L temperature logger had a measurement range of −40 to 85 °C and an accuracy of ±0.5 °C within the calibrated temperature range of −10 to 65 °C. The BENETECH GM1365 data logger had a temperature measurement range of −30 to 80 °C and an accuracy of ±0.3 °C within the range of 10–60 °C. Its relative-humidity measurement range was 0–100%, with an accuracy of ±2%. The Fluke TiS55+ thermal camera had a temperature measurement range of −20 to 550 °C, although measurements below −10 °C were not calibrated. The stated temperature accuracy was ±2 °C at an ambient temperature of 25 °C. The camera simultaneously recorded infrared and visible-light images, and the infrared detector resolution was 256 × 192 pixels.

#### 2.2.2. Data-Collection Procedure

Rectal temperature was used as an approximation of core body temperature. Following defecation by an elephant selected for monitoring, an iButton DS1922L temperature logger, approximately 17.3 mm in diameter and 6 mm thick, was lubricated with petroleum jelly and inserted approximately 30 cm into the rectum. The temperature logger was programmed to record temperature at 5 min intervals. At the elephant’s next defecation, generally 1–3 h later, the device was expelled with the feces. It was then recovered, disinfected, cleaned, and connected to the associated software to retrieve the recorded rectal-temperature data. After rectal-temperature monitoring began, ambient temperature, relative humidity, and body-surface temperature were recorded synchronously according to the 5 min recording interval programmed into the iButton logger. Ambient temperature and relative humidity were measured using the BENETECH GM1365 data logger, which was positioned on a support approximately 10 cm above the ground at the elephant enclosure. The device was placed in a location protected from direct solar radiation. Infrared thermograms were obtained from one side of each elephant using the Fluke TiS55+ thermal camera at a distance of approximately 5 m. The reflected background temperature setting of the thermal camera was adjusted to the measured ambient air temperature. Emissivity was set to 0.98, a value widely adopted in infrared thermography studies of elephants and other large sparsely haired pachyderms [14].

During field data collection, the eight elephants were monitored repeatedly, yielding 425 temporally matched sets of rectal temperature, body-surface temperature, ambient temperature, and relative humidity measurements, each paired with a corresponding infrared thermogram. All 425 contemporary thermograms were linked to confirmed individual elephant identities. In addition to the contemporary dataset, historical infrared thermograms collected by the same research group at the same study site were incorporated to expand the dataset for model training. These images, acquired between March 2016 and January 2020 and November 2023 and October 2024, cover semi-captive, captive, and wild Asian elephants. All contemporary and historical thermograms were captured using identical camera hardware, resolution, calibration procedures, emissivity settings, imaging distances, and standard operating protocols, by field personnel trained under a unified protocol within the research group. Individual identity was not systematically annotated for the historical images, so the exact total number of unique individuals across the full 2372-image dataset cannot be determined; however, it was verified that none of the eight semi-captive elephants from the contemporary cohort were represented in the historical image pool.

All available historical thermograms underwent preliminary screening followed by random sampling to form the final expanded dataset. Images were excluded based on the following pre-defined criteria: (1) severe motion blur or defocus; (2) imaging angle deviating by more than 45° from the lateral view; (3) imaging distance outside the 4.5–5.5 m range; (4) more than 50% of the outer auricle obscured by vegetation, conspecifics, or enclosure structures. Following screening, stratified random sampling was applied to eligible historical images to balance representation across elephant management types (captive, semi-captive, wild) and field seasons. In total, 2372 unprocessed infrared thermograms (425 contemporary + 1947 historical) were retained for subsequent dataset construction.

### 2.3. Selection of the Body Region for Automated Detection

**Figure 1:**
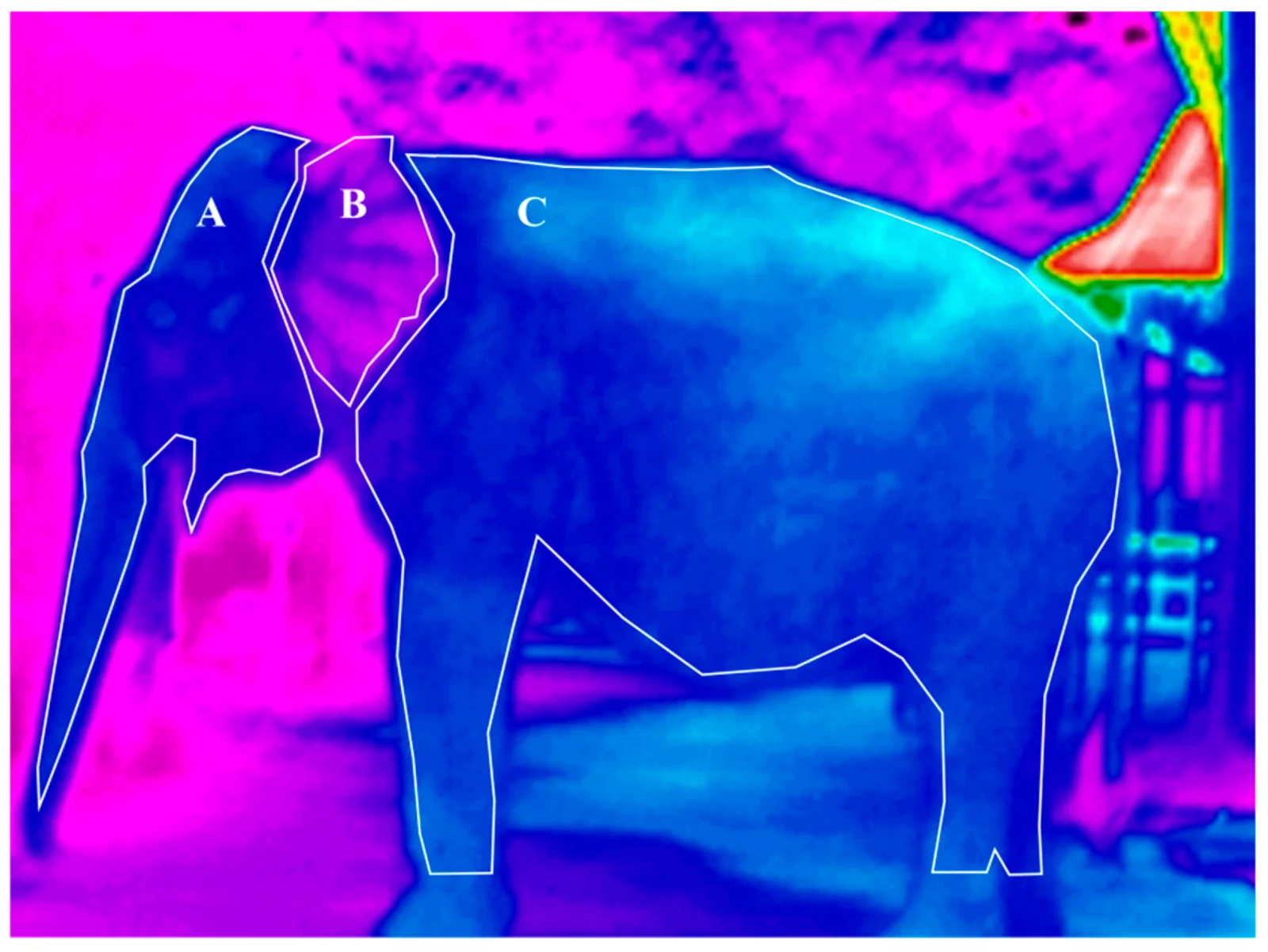
Example of the anatomical regions delineated on an infrared thermogram of an Asian elephant. (A) Head; (B) auricle; and (C) torso and limbs.

The body surface of each Asian elephant was divided into three anatomical regions using Fluke Connect software (version 2.0.10.0): the head, the outer ear, and the torso-and-limb region, as illustrated in Figure 1. Surface-temperature values for the corresponding regions were extracted directly using the software.

Both manual region delineation and automated object detection may introduce measurement error when the selected region includes pixels representing the surrounding background. Because the surface temperatures of the elephants were observed to remain higher than the ambient background temperature across seasons, both the maximum and mean temperatures within each delineated region were used to characterize regional surface temperature.

Because repeated observations came from only eight elephants and were statistically non-independent, repeated-measures correlation (*rmcorr*) was applied to assess within-individual linear associations between regional surface temperature and rectal temperature. This method accounts for non-independence of repeated samples by removing individual-level baseline offsets, avoiding the pseudoreplication problem of conventional Pearson/Spearman correlation. The anatomical region yielding the largest magnitude of r_rm_ (repeated-measures correlation coefficient) was pre-defined as the target for automated detection.

### 2.4. Dataset Construction

The outer auricular region was manually annotated on each infrared thermogram using the LabelImg image annotation tool (version 1.8.6). All annotations were completed by a single trained annotator to eliminate inter-rater variability. The bounding box for the outer auricle was defined according to consistent anatomical criteria: the box fully enclosed the entire cartilaginous pinna from the apical tip at the upper edge to the ear base at the lower edge, and from the cutaneous junction with the head medially to the free lateral edge of the ear. All annotations were stored in YOLO format for subsequent model training and intrinsic detection performance evaluation. From the full set of 2372 annotated thermograms, the dataset was partitioned into training, validation, and external test sets in an approximate 9:1:1 ratio, consisting of 1974, 204, and 194 images, respectively. The validation and test sets were both sourced exclusively from the 425 contemporary thermograms collected in this study and were split via individual-stratified sampling to ensure no overlap in elephant identity between the two subsets. The remaining contemporary images not allocated to the validation or test sets were combined with all historical thermograms to form the training set. Critically, the external test set contained images from distinct Asian elephant individuals that were not represented in either the training or validation sets, providing strictly independent evaluation for automated temperature extraction performance.

### 2.5. Automated Detection of the Outer Ear and Temperature Extraction

#### 2.5.1. Selection of YOLO Architectures and Model Sizes

Infrared thermography was combined with deep learning to automatically detect the outer auricular region and extract its corresponding surface temperature.

The You Only Look Once family of object-detection algorithms is based on deep convolutional neural networks and provides an end-to-end, single-stage framework for real-time object detection [32,33]. YOLO models formulate object detection as a unified regression problem and directly predict bounding-box coordinates and class probabilities from the complete input image. This design enables rapid and accurate object localization and classification, with low inference latency and scalable network architectures [33].

YOLO architectures are commonly available in five model sizes: nano (n), small (s), medium (m), large (l), and extra-large (x). Smaller models, particularly the n and s variants, generally provide faster inference and lower computational requirements, whereas larger models may achieve greater detection accuracy at the cost of increased computational demand. Automated wildlife-monitoring systems may ultimately need to be deployed on edge-computing devices positioned close to the source of image acquisition. Edge-based processing reduces the need to transmit large image datasets to cloud servers, lowers communication latency, improves data privacy, and may be particularly valuable in remote field settings with limited network connectivity.

To balance detection accuracy, inference speed, and potential deployment under constrained computational conditions, four YOLO generations were selected: YOLOv5, YOLOv8, YOLO11, and YOLO26. The two lightest model sizes, n and s, were evaluated for each generation. The resulting eight models were: YOLOv5n; YOLOv5s; YOLOv8n; YOLOv8s; YOLO11n; YOLO11s; YOLO26n; and YOLO26s. All eight models were trained and tested in parallel to compare their ability to detect the outer ear in elephant infrared thermograms and to identify the model most suitable for automated auricular temperature extraction and potential real-time deployment.

#### 2.5.2. Model Training Details

To ensure reproducibility and enable fair comparison across all model variants, the eight YOLO models were trained under a unified computing environment and consistent hyperparameter configurations.

All training and inference experiments were conducted on the same hardware platform: an AMD Ryzen 9 5900HS central processing unit (Advanced Micro Devices, Inc. (AMD), Santa Clara, CA, USA), an NVIDIA GeForce RTX 3060 graphics processing unit with 6 GB dedicated video memory (NVIDIA Corporation, Santa Clara, CA, USA), and 16 GB of system memory, running the Windows 11 operating system. Software dependencies included CUDA 11.5 for GPU-accelerated computation, Python 3.8.20, and the PyTorch 2.0.1 deep learning framework. All model implementations and training pipelines were built on the Ultralytics YOLO framework. Key training hyperparameters were standardized across all eight model variants. Input images were resized to a uniform resolution of 640 × 640 pixels. The batch size was fixed at 16, with a maximum training limit of 80 epochs. A stochastic gradient descent (SGD) optimizer was adopted with a momentum of 0.937 and a weight decay of 0.0005. The initial learning rate was set to 0.01, and a cosine annealing learning rate scheduler was applied to dynamically adjust the learning rate over the training process, with a final learning rate ratio of 0.01. All models were initialized with weights pre-trained on the COCO dataset to leverage transfer learning and stabilize convergence. An early stopping mechanism was enabled to mitigate overfitting: training was automatically terminated if validation loss showed no improvement for 15 consecutive epochs. A fixed random seed (seed = 42) was specified for all training runs to guarantee full reproducibility of results. Model performance was evaluated on the validation set after each epoch to monitor convergence and trigger the early stopping callback.

A standard data augmentation pipeline was applied during training to improve model generalization and robustness. Geometric augmentations included random horizontal flipping, random scaling and translation, and mosaic augmentation that stitched four training samples into one composite image. HSV color-space perturbations were also introduced to accommodate variations in pseudo-color rendering of thermal images. All augmentation operations were restricted to the object detection training phase; quantitative temperature extraction was performed on raw unmodified thermograms to preserve measurement accuracy.

#### 2.5.3. Automated Extraction of Outer-Ear Temperature

When thermograms were recorded using the Fluke TiS55+ thermal camera, the instrument simultaneously stored the original temperature matrix associated with each image. Each element of this matrix corresponded to the temperature value of an individual pixel in the infrared image. For each image, the YOLO-based detector generated the bounding-box coordinates of the detected outer-ear region: (x1, y1, x2, y2). These coordinates were mapped to the corresponding row and column indices of the temperature matrix. All pixel-level temperature values within the detected bounding box were then extracted, and the mean and maximum temperatures of the outer-ear region were calculated.

### 2.6. Model Evaluation and Selection

The eight YOLO models were evaluated at two complementary levels: intrinsic object-detection performance, and agreement between automatically and manually extracted temperature values. The results from these two levels were combined to select the model that provided the most appropriate balance between target detection, temperature-extraction agreement, and computational efficiency.

#### 2.6.1. Intrinsic Detection Performance

Intrinsic detection performance represented the ability of each model to identify and localize the outer auricular region. Four evaluation metrics were used: precision, recall, F1 score, and mean Average Precision at an intersection-over-union threshold of 0.50 (mAP50).

Precision and recall were calculated as:

(1) $$P_{r}=\frac{TP}{TP+FP}$$

(2) $$R_{c}=\frac{TP}{TP+FN}$$

where TP represents true-positive detections, FP represents false-positive detections and FN represents false-negative detections.

The F1 score was calculated as the harmonic mean of precision and recall and was used to evaluate the balance between these two metrics; it was calculated as:

(3) $$F1=\frac{2P_{r}R_{c}}{P_{r}+R_{c}}$$

AP (Average Precision) summarizes the precision–recall curve by computing the area under the curve for a specific class. mAP (mean Average Precision) is the average of the AP values across all object classes. In this study, mAP50 was used to represent the area under the precision–recall curve when a predicted bounding box was considered correct at an intersection-over-union threshold of 0.50.

(4) $$AP=\int_{0}^{1}P(r)dr$$

(5) $$mAP=\frac{1}{k}\sum_{i=1}^{k}AP_{i}$$

Inference speed was expressed as frames processed per second (FPS) and was included as an indicator of the potential suitability of each model for real-time monitoring.

#### 2.6.2. Evaluation of Temperature-Extraction Agreement

Intrinsic object-detection metrics quantify target localization but do not directly demonstrate whether the model accurately extracts temperature from the selected anatomical region. Temperature-extraction agreement was therefore evaluated by comparing temperatures obtained from YOLO-generated bounding boxes with those extracted from manually delineated reference regions using Fluke Connect software.

Each trained YOLO model was applied to the independent test images. The proportion of test images in which the outer ear was successfully detected was recorded as the detection rate R. The mean temperature and the maximum temperature of all pixels within the bounding box were selected as two temperature variables for evaluation. For each model and temperature variable, agreement between automated and manual measurements was assessed using Bland–Altman analysis and Taylor diagrams. Bland–Altman analysis calculated the mean difference, or bias, between automated and manually extracted temperatures and the corresponding 95% limits of agreement: (LoA = bias ± 1.96 × standard deviation). Bias approaching zero and narrow limits of agreement indicated stronger agreement between automated and manual temperature extraction. Taylor diagrams used manually extracted temperature as the reference and summarized three statistics: the correlation coefficient (*r*); the standard deviation (σ); and the centered root-mean-square difference (RMSD). The ideal model was expected to lie close to the reference point, corresponding to *r* = 1, *σ* = *σ*_ref, and RMSD = 0.

The combined use of conventional detection metrics and direct temperature-agreement analysis is important because highly accurate anatomical localization does not necessarily guarantee equally accurate extraction of thermal values [25,27].

#### 2.6.3. Weighted Multi-Criteria Model Selection

Intrinsic detection performance and temperature-extraction agreement evaluated different aspects of model performance. A single metric was therefore considered insufficient for model selection. For example, a model with a high mAP50 could still produce relatively large temperature bias, whereas a model with a high correlation coefficient could fail to detect the outer ear in a substantial proportion of images, rendering temperature extraction infeasible.

A weighted multi-criteria model-selection framework was therefore developed to combine the different performance dimensions into a single quantitative score. Seven indicators were included: detection rate; absolute temperature bias; standard-deviation ratio; correlation coefficient; centered RMSD; F1 score; and mAP50. Weights were assigned based on the core objective of practical auricular temperature monitoring in Asian elephants, combined with expert consensus in veterinary infrared thermography and established conventions from comparable automated wildlife monitoring studies. Full weight assignments are provided in Table 1.

A two-stage pre-defined decision rule was applied for final model selection. First, weighted composite scores were calculated separately for mean-temperature and maximum-temperature extraction tasks to rank model performance on each thermal metric. Second, the final optimal model was determined by jointly considering three pre-specified criteria: (1) cross-task ranking stability across both mean and maximum temperature extraction; (2) real-time inference speed; and (3) suitability for potential edge deployment in field monitoring scenarios. All selection criteria were specified prior to model training and evaluation.

To assess the robustness of the final model ranking and rule out potential bias from subjective weight allocation, a Monte Carlo weight perturbation analysis was conducted. Each of the seven indicator weights was randomly varied within a ±20% range around its baseline value, generating 1000 independent weight sets. Each perturbed weight set was renormalized to preserve a constant total sum of weights, ensuring consistent scoring scale across all iterations. The composite score and corresponding rank of each candidate model were recalculated for every weight set.

Because the indicators had different scales and optimal directions, they were normalized to the interval from 0 to 1, with higher normalized scores representing better performance.

For positively oriented indicators, including detection rate (R) and correlation coefficient (r), the normalized score was calculated as:

(6) $$S_{i}=\frac{X_{i}-X_{min}}{X_{\max}-X_{min}}$$

For negatively oriented indicators, including the absolute bias and RMSD, the normalized score was calculated as:

(7) $$S_{i}=1-\frac{X_{i}-X_{min}}{X_{\max}-X_{min}}$$

For the standard-deviation ratio, for which the optimal value was 1, the normalized score was calculated as:

(8) $$S_{i}=1-\frac{\left|\sigma_{\mathrm{ratio},i}-1\right|}{\max_{j}\left(\left|\sigma_{\mathrm{ratio},i}-1\right|\right)}$$

where Xi represents the original value of the relevant indicator for model i, and Xmin and Xmax represent the minimum and maximum values observed among the eight models.

The overall weighted score was calculated as:

(9) $$S_{\mathrm{total}}=0.2S_{\det}+0.15S_{bias}+0.15S_{corr}+0.15S_{std}\;+0.15S_{RMSD}+0.1S_{F1}+0.1S_{mAP50}$$

where $S_{\det}$, $S_{bias}$, $S_{corr}$, $S_{std}$, $S_{RMSD}$, $S_{F1}$ and $S_{mAP50}$ represent the normalized scores for detection rate, absolute bias, correlation coefficient, standard-deviation ratio, centered RMSD, F1 score, and mAP50, respectively.

The eight models were ranked from the highest to the lowest overall score. The model with the highest score was selected as the preferred model for automated outer-ear detection and temperature extraction.

## 3. Results

### 3.1. Selection of the Optimal Thermal Monitoring Region

Repeated-measures correlation was computed for each regional temperature metric and environmental variable against rectal temperature. All tested variables showed statistically significant within-individual associations (df = 417, all *p* < 0.001).

Among anatomical-region derived metrics (Table 2), mean outer-ear temperature (ET_avg_) yielded the largest within-individual correlation with rectal temperature (*r_rm_* = 0.395, 95%CI [0.30, 0.48]), followed by mean head temperature (*r_rm_* = 0.392, 95%CI [0.30, 0.48]) and mean torso-and-limb temperature (*r_rm_* = 0.381, 95%CI [0.29, 0.47]). For maximum-temperature metrics across regions, *r_rm_* values were comparatively weaker: ET_max_ *r_rm_* = 0.362, HT_max_ *r_rm_* = 0.331, TLT_max_ *r_rm_* = 0.307.

Following our pre-specified selection criterion, mean outer-ear temperature (ET_avg_) was selected as the target metric for subsequent automated detection and temperature-extraction workflows.

### 3.2. Automated Detection of the Outer Auricular Region

**Figure 2:**
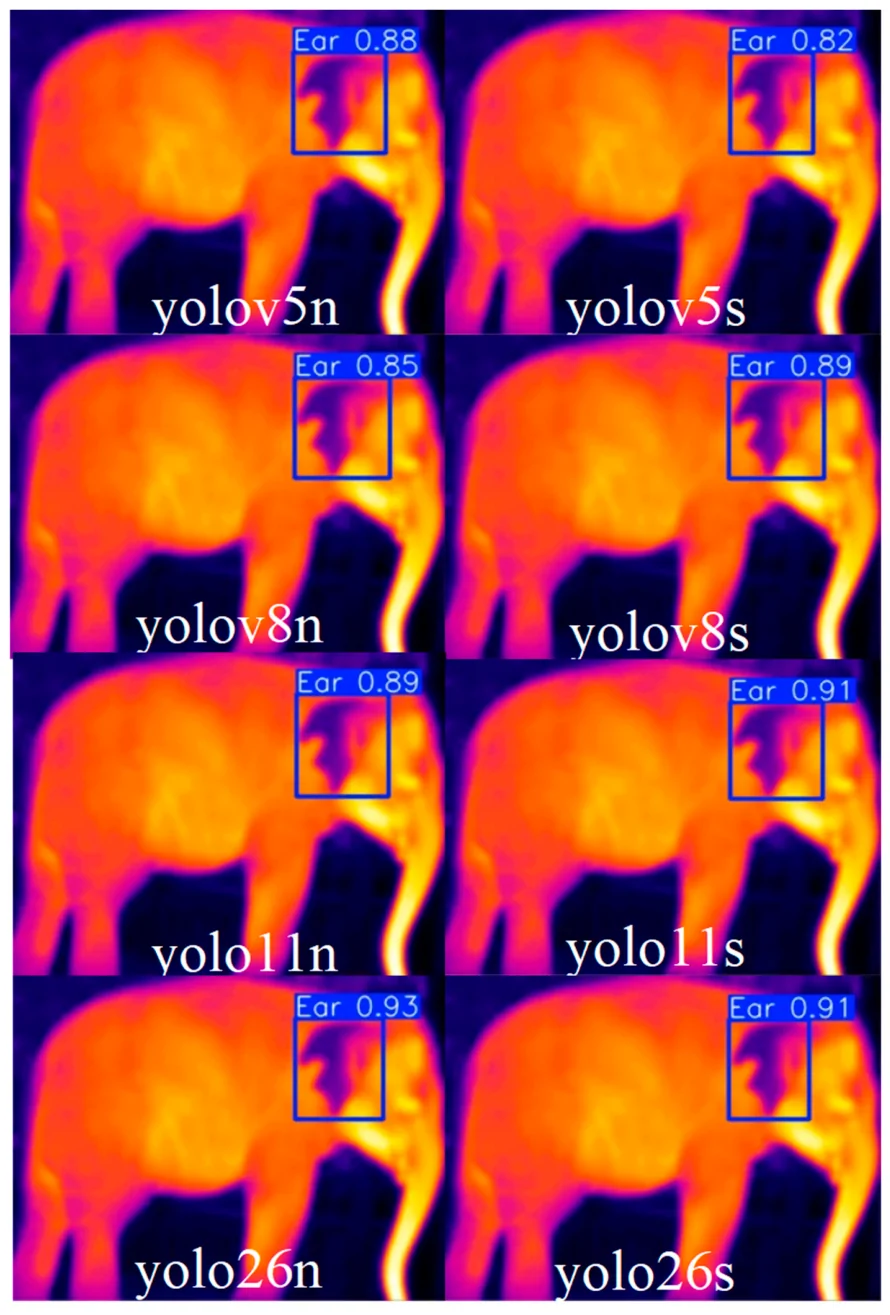
Representative automated detections of the outer auricular region of an Asian elephant using eight YOLO models: YOLOv5n, YOLOv5s, YOLOv8n, YOLOv8s, YOLO11n, YOLO11s, YOLO26n, and YOLO26s. The bounding boxes indicate the detected outer-ear region, and the associated values represent model confidence scores.

Representative detection results obtained using the eight trained models—YOLOv5n, YOLOv5s, YOLOv8n, YOLOv8s, YOLO11n, YOLO11s, YOLO26n, and YOLO26s—are shown in Figure 2. All models successfully identified the outer auricular region in infrared thermograms with a resolution of 256 × 192 pixels, although differences were observed in detection confidence and bounding-box placement.

The intrinsic detection performance of the eight models on the validation dataset is presented in Table 3. Overall, all models achieved satisfactory precision and recall. YOLOv8s showed the strongest combined precision–recall performance, with a precision of 92.9%, a recall of 92.4%, and an F1 score of 92.6%. YOLOv8n and YOLO11n also performed well, whereas YOLOv5n produced the lowest precision and F1 score among the evaluated models.

All eight models achieved an mAP50 of at least 0.920, indicating generally strong detection of the elephant outer ear. YOLO26s obtained the highest mAP50 value of 0.947, followed by YOLOv8s at 0.940. YOLOv5s had the lowest mAP50, at 0.920. Within most YOLO generations, the larger s models achieved slightly higher mAP50 values than the corresponding n models, indicating a modest detection-accuracy advantage associated with increased model capacity. Inference speed showed the opposite general pattern. The lighter n models were faster than the corresponding s models. YOLOv5n achieved the highest inference speed, processing 213 frames per second (FPS), followed by YOLO26n at 189 FPS and YOLO11n at 164 FPS. Among the s models, YOLOv5s, YOLO11s, and YOLOv8s achieved similar inference speeds of 108, 110, and 99 FPS, respectively, whereas YOLO26s was the slowest model, at 53 FPS. These results indicate that the n models provided a clear computational-efficiency advantage and may therefore be better suited to real-time processing or deployment on devices with limited computational resources.

### 3.3. Automated Temperature Extraction and Agreement Assessment

The intrinsic detection metrics described above—including precision, recall, F1 score, and mAP50—quantified the ability of each model to identify and localize the outer ear. However, these metrics did not directly indicate whether the resulting bounding boxes enabled reliable extraction of auricular temperature. The trained YOLO models were therefore combined with the original infrared temperature matrices to extract temperature values from the detected outer-ear regions in the external test dataset. Temperatures extracted from manually delineated regions using Fluke Connect software were used as the reference measurements. It should be clarified that these manual measurements served as the reference method for region delineation and temperature extraction, rather than representing absolute ground truth of true auricular tissue temperature. Manual region delineation is subject to minor variability in boundary judgment even among trained operators, and infrared thermography measures surface radiative temperature rather than deep tissue temperature, which is inherently influenced by emissivity settings and reflected ambient radiation. Accordingly, the agreement analyses below quantify the interchangeability between automated and manual workflows, rather than the absolute accuracy of temperature values relative to a theoretical true temperature. For each model, two thermal variables were evaluated: the mean temperature of all pixels within the detected region and the maximum temperature within the detected region. Agreement between automated and manually extracted values was evaluated using Bland–Altman analyses and Taylor diagrams.

#### 3.3.1. Agreement in Mean Outer-Ear Temperature

**Figure 3:**
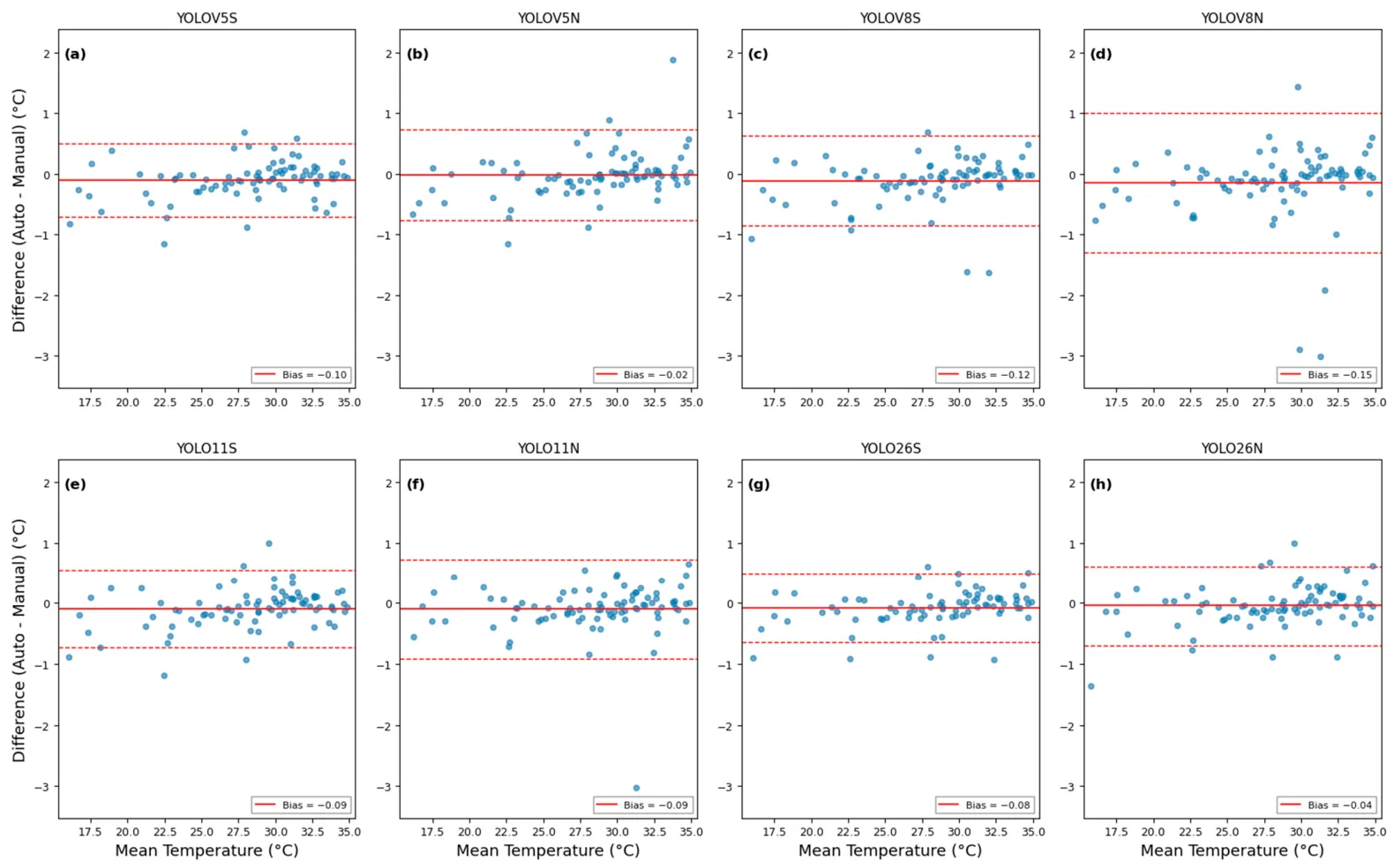
Bland–Altman plots comparing manually and automatically extracted mean outer-ear temperatures for the eight YOLO models. (**a**) YOLOv5s; (**b**) YOLOv5n; (**c**) YOLOv8s; (**d**) YOLOv8n; (**e**) YOLO11s; (**f**) YOLO11n; (**g**) YOLO26s; (**h**) YOLO26n. The solid horizontal line represents the mean difference, or bias, between the automated and manual measurements. The dashed horizontal lines represent the 95% limits of agreement.

Figure 3 presents the Bland–Altman plots comparing automatically and manually extracted mean outer-ear temperatures for the eight YOLO models.

The absolute mean bias was below 0.15 °C for all models, indicating little systematic overestimation or underestimation of the manually extracted mean temperature. YOLOv5n showed the smallest bias, at −0.02 °C, followed by YOLO26n, at −0.04 °C. Differences among the models were more evident in the widths of the 95% limits of agreement. YOLO26s produced the narrowest limits of agreement, ranging from −0.641 to 0.489 °C, corresponding to a total interval width of approximately 1.13 °C. This result indicated the strongest agreement and greatest stability in mean-temperature extraction among the eight models. YOLOv5s and YOLO11s also showed relatively narrow limits of agreement. The limits were −0.707 to 0.503 °C for YOLOv5s and −0.721 to 0.548 °C for YOLO11s. Detailed results of the Bland–Altman analysis are presented in Table S2.

#### 3.3.2. Agreement in Maximum Outer-Ear Temperature

**Figure 4:**
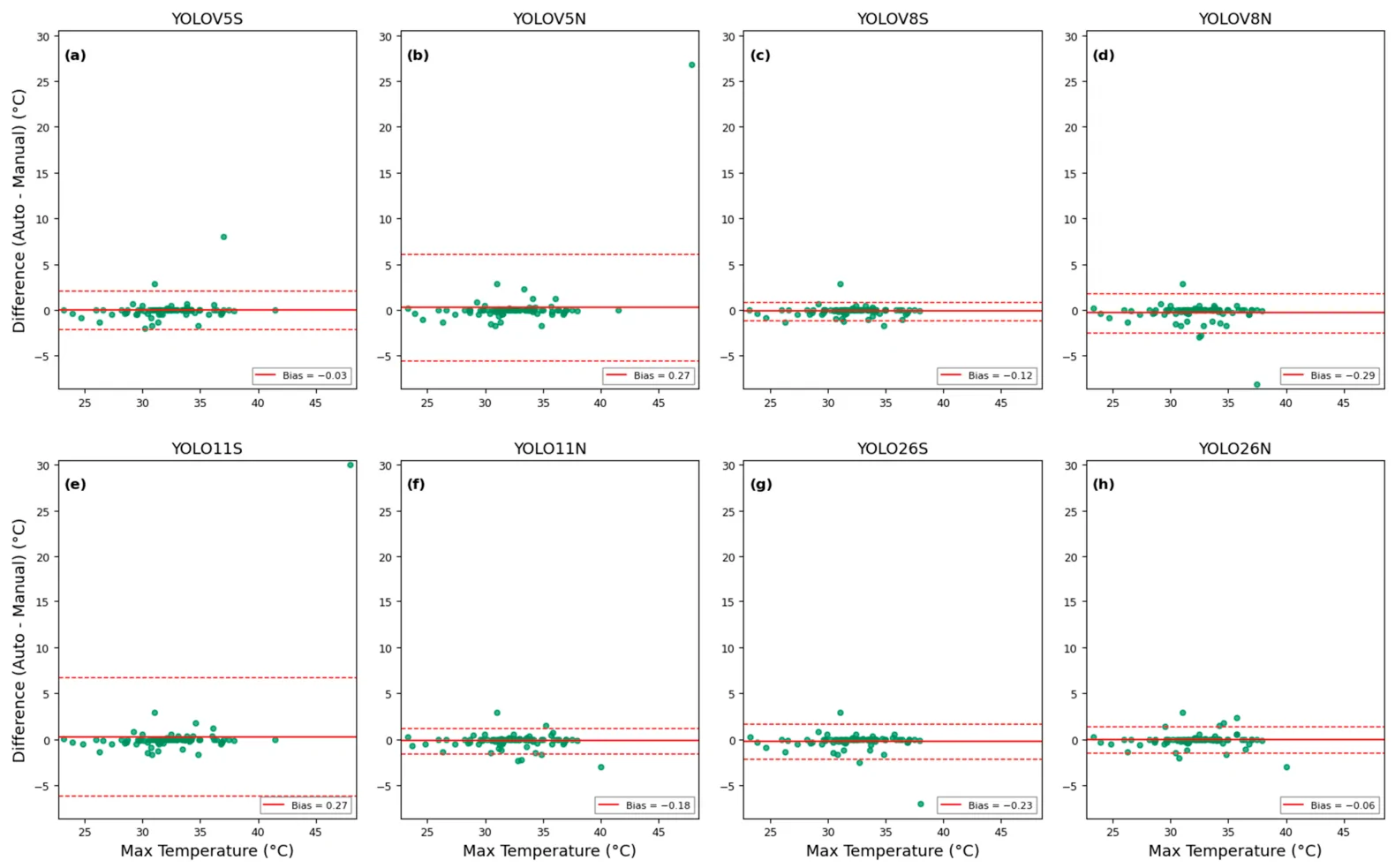
Bland–Altman plots comparing manually and automatically extracted maximum outer-ear temperatures for the eight YOLO models. (**a**) YOLOv5s; (**b**) YOLOv5n; (**c**) YOLOv8s; (**d**) YOLOv8n; (**e**) YOLO11s; (**f**) YOLO11n; (**g**) YOLO26s; (**h**) YOLO26n. The solid horizontal line represents the mean difference, or bias, between automated and manual measurements. The dashed horizontal lines represent the 95% limits of agreement.

Figure 4 presents the Bland–Altman plots comparing automatically and manually extracted maximum outer-ear temperatures.

In general, the biases and limits of agreement were larger for maximum-temperature extraction than for mean-temperature extraction. YOLOv8s showed the smallest bias for maximum temperature, at −0.125 °C. YOLO26s and YOLOv8n had biases of −0.227 and −0.295 °C, respectively.

The differences among models were particularly pronounced in the 95% limits of agreement. YOLOv8s produced the narrowest interval, ranging from −1.138 to 0.889 °C, with a total width of approximately 2.03 °C. This result indicated that YOLOv8s provided the strongest agreement between automated and manual extraction of maximum outer-ear temperature. By contrast, YOLOv5n and YOLO11s showed substantially wider limits of agreement. The interval for YOLOv5n ranged from −5.535 to 6.069 °C, corresponding to a width of approximately 11.60 °C. For YOLO11s, the interval ranged from −6.190 to 6.736 °C, corresponding to a width of approximately 12.93 °C. These wide intervals indicated considerable variability in maximum-temperature extraction by these two models. Detailed results of the Bland–Altman analysis are presented in Table S2.

Within several YOLO generations, the s model produced narrower limits of agreement than the corresponding n model. This pattern suggests that the larger models may have provided more stable temperature extraction in some model generations, although the relationship between model size and extraction performance was not uniform across all evaluated architectures.

#### 3.3.3. Taylor Diagram Evaluation

**Figure 5:**
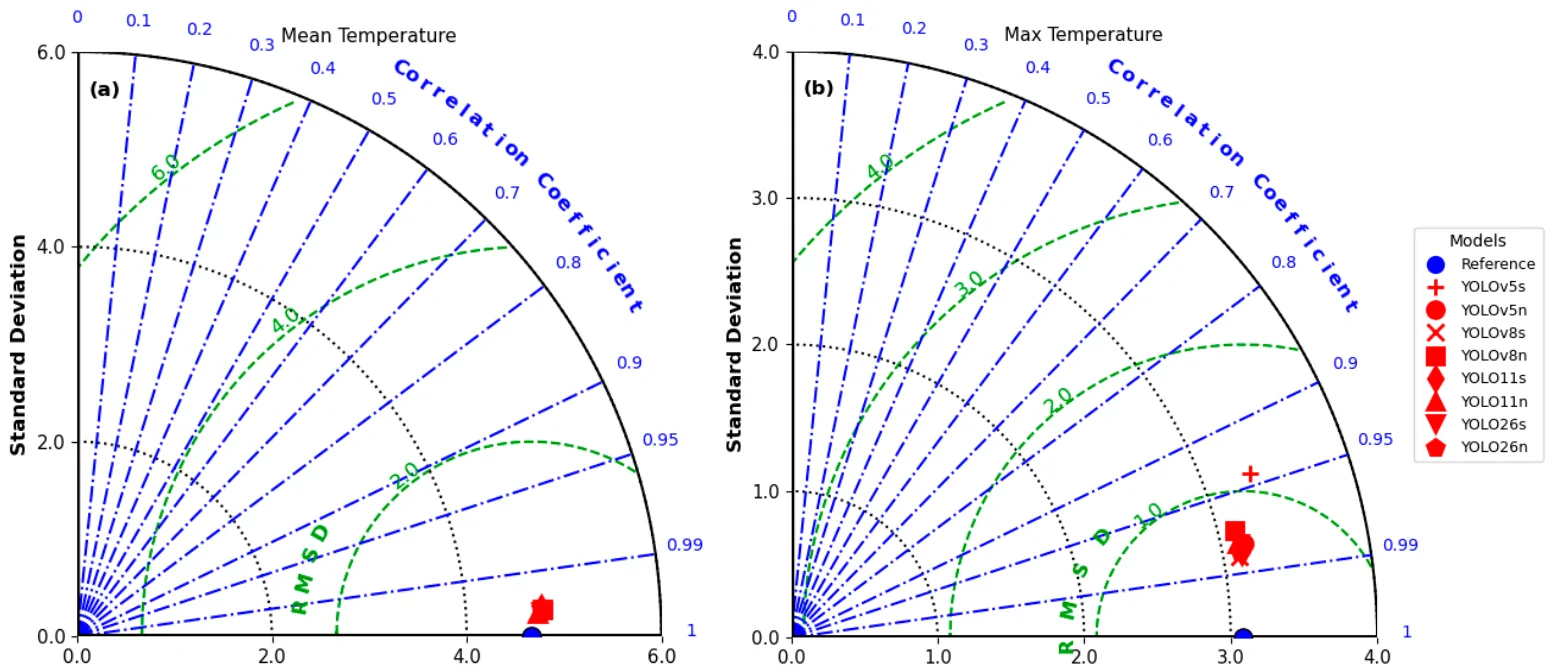
Taylor diagrams comparing manually and automatically extracted Asian elephant outer-ear temperatures. (**a**) Mean outer-ear temperature; (**b**) maximum outer-ear temperature. The blue circle represents the manually extracted reference values. Models positioned closer to the reference point show stronger agreement with the reference measurements.

Taylor diagrams were used to integrate the correlation coefficient, standard deviation, and centered root-mean-square difference into a single graphical comparison of model-derived and manually extracted temperatures (Figure 5).

For mean-temperature extraction, all models were positioned close to the reference point in the Taylor diagram, indicating strong agreement with the manually extracted values. YOLO11n showed the strongest overall performance, with a correlation coefficient of 0.9986, a standard-deviation ratio of 1.014, and an RMSD of 0.262 °C. YOLO26n ranked closely behind, with a correlation coefficient of 0.9986, a standard-deviation ratio of 1.022, and an RMSD of 0.282 °C. Greater separation among models was observed for maximum-temperature extraction. YOLOv8s was closest to the reference point, with a correlation coefficient of 0.984, a standard-deviation ratio of 1.009, and an RMSD of 0.565 °C. YOLOv5s was positioned farthest from the reference point according to the values reported in the original analysis, with a correlation coefficient of 0.942, a standard-deviation ratio of 1.079, and an RMSD of 1.115 °C. Detailed results of the Taylor diagram analysis are presented in Table S3.

Overall, the Taylor diagram findings were broadly consistent with the Bland–Altman analyses. Together, the two approaches identified YOLOv8s and YOLO11n as leading candidates for automated temperature extraction, although their relative performance differed between mean- and maximum-temperature measurements.

### 3.4. Weighted Multi-Criteria Model Ranking

Following the pre-defined two-stage model selection framework described in Section 2.6.3, weighted composite scores were first computed separately for mean and maximum temperature extraction tasks, and cross-task stability and inference speed were then jointly considered to identify the final optimal model.

Seven indicators were normalized and combined for each model: detection rate, absolute bias, standard-deviation ratio, correlation coefficient, centered RMSD, F1 score, and mAP50. The resulting model rankings and composite scores are presented in Table 4.

For mean-temperature extraction, YOLO11n achieved the highest composite score, at 0.727. YOLOv8n ranked second, with a score of 0.628, followed by YOLO26s, with a score of 0.445. YOLOv5s obtained the lowest score, at 0.196. For maximum-temperature extraction, YOLOv8n achieved the highest composite score, at 0.861. YOLO26n ranked second, with a score of 0.815, followed by YOLO11n, with a score of 0.776. YOLOv5s again ranked last, with a score of 0.157. YOLOv5s ranked last for both mean- and maximum-temperature extraction, indicating comparatively weak overall performance for the automated outer-ear temperature-extraction task. YOLOv5n also ranked near the bottom in both evaluations. YOLO11n showed the most stable cross-task performance. It ranked first for mean-temperature extraction and third for maximum-temperature extraction. YOLOv8n also performed consistently, ranking second for mean temperature and first for maximum temperature. To evaluate the robustness of our model selection against subjective weight assignments, we conducted a sensitivity analysis by perturbing the seven indicator weights using a Monte Carlo approach. Each weight was randomly varied within ±20% of its baseline value, generating 1000 renormalized weight sets for which model rankings were recomputed. As shown in Table S4, for mean temperature, YOLO11n ranked first in 100% of the 1000 perturbations, while YOLOv8n consistently ranked second under the same conditions. For maximum temperature, YOLOv8n consistently ranked first in all perturbations, whereas YOLO11n ranked within the top three in 64.6% of the cases.

Notably, the lightweight n models generally ranked higher than the corresponding s models in the weighted evaluation. This pattern differed from the conventional expectation that larger models should consistently provide better performance. The results indicate that temperature-extraction performance was not determined solely by conventional object-detection accuracy. Consistent bounding-box placement may be particularly important when thermal values are extracted directly from the pixels enclosed by the predicted region.

Considering the rankings for both mean and maximum temperature, together with inference speed and potential deployment requirements, YOLO11n was selected as the preferred model for automated outer-ear temperature extraction. YOLO11n ranked first for mean-temperature extraction, with a composite score of 0.727; it ranked third for maximum-temperature extraction, with a composite score of 0.776; it showed strong performance across both temperature measures; and its inference speed of 164 FPS was substantially higher than those of YOLOv8s at 99 FPS and YOLO26s at 53 FPS. YOLO11n was therefore selected for subsequent system integration and potential real-time deployment.

## 4. Discussion

### 4.1. Thermoregulatory Significance of the Outer Ear in Asian Elephants

In the present study, outer-ear temperature showed the strongest association with rectal temperature among the three investigated body regions, with mean outer-ear temperature exhibiting a moderate positive correlation. This finding supports the use of the auricle as an informative region for non-invasive monitoring of thermal status changes in Asian elephants, though it does not indicate that auricular temperature is equivalent to core body temperature or can serve as a direct substitute without further predictive validation.

As specialized body-surface regions facilitating heat exchange between animals and their surroundings, thermal windows are generally characterized by dense superficial vascularization, relatively limited insulation, and the capacity for physiological regulation of peripheral blood flow; some studies define temporary thermal windows as localized surface regions where temperature differs from adjacent areas by more than approximately 5 °C [34]. The elephant auricle is particularly well suited to this thermoregulatory role: it has a high surface-area-to-volume ratio, sparse hair, and an extensive vascular network, and ear-flapping movements promote both convective and radiative heat loss by repeatedly exposing the inner auricular surface to airflow [35], enabling blood circulating through the superficial vessels of the ear to transfer internal heat to the surrounding environment.

Previous infrared thermographic studies have provided consistent evidence for this function. Weissenböck et al. examined six African elephants maintained at outdoor temperatures of −4.1 to 7.5 °C and indoor temperatures of 15.0 to 20.3 °C, observing thermal windows in 43.9% of measurements and noting considerable variation in the size, shape, and temperature of auricular thermal windows [36]. Lefebvre et al. investigated thermoregulatory patterns in 10 Asian elephants exposed to low winter temperatures and found thermal windows occurred primarily on the ears, with their occurrence and distribution associated with individual age, though no significant relationship between ambient temperature and thermal window appearance was detected under the cold conditions examined [37]. Studies of physical activity have further highlighted the ear’s thermoregulatory role: Rowe et al. demonstrated that Asian elephants accumulate substantial body heat during submaximal exercise and rely on behavioral mechanisms to manage the thermoregulatory constraints associated with their large body size [15], and Tilley et al. subsequently reported marked regional surface-temperature changes in active Asian elephants, with the ears showing particularly important thermal responses [16].

The present results extend these observations by showing that outer-ear temperature remains associated with rectal temperature under the relatively moderate resting conditions of this study, indicating that the ear functions not only as a site of active heat dissipation during exercise or extreme environmental exposure, but also as a potentially informative thermal region during routine physiological monitoring. Nevertheless, the association between auricular and rectal temperature is moderate rather than sufficiently strong for direct estimation of core temperature, so outer-ear temperature should be interpreted as a peripheral thermal indicator whose relationship with core temperature is influenced by vascular regulation, environmental conditions, behavior, and individual characteristics.

### 4.2. Differences in Model Performance

The eight YOLO models generally achieved strong intrinsic detection performance. Within several model generations, the larger s variants obtained slightly higher mAP50 values than the corresponding n variants. For example, YOLOv8s achieved an mAP50 of 0.940, compared with 0.936 for YOLOv8n. This pattern was broadly consistent with the expectation that models with greater parameter capacity may achieve improved object-localization performance.

However, the weighted rankings based on temperature extraction produced a different pattern. YOLO11n ranked first for mean-temperature extraction, while YOLOv8n ranked second. For maximum-temperature extraction, YOLOv8n ranked first and YOLO11n ranked third. The lighter n models therefore generally performed better than the corresponding s models in the multi-criteria evaluation.

This result indicates that conventional object-detection accuracy and temperature-extraction performance are not necessarily equivalent. A model may generate a bounding box that satisfies the intersection-over-union threshold used for mAP calculation while still including or excluding pixels that materially affect the extracted temperature. This issue is particularly important for maximum-temperature extraction because the result may be determined by only one or a small number of high-temperature pixels.

Previous automated animal-temperature studies have similarly demonstrated the need to evaluate both anatomical detection and the temperatures extracted from the detected regions [24,25,26]. Xie et al. combined deep-learning-based anatomical detection with temperature extraction in pigs and showed that the practical value of the model depended on both localization and thermal measurement performance [24]. Zhang et al. also used direct agreement analyses when evaluating automated extraction of sheep eye temperature [26].

One possible explanation for the performance observed in the present study is that some lighter models produced more consistent bounding-box placement, even when their conventional mAP values were marginally lower. In contrast, a more complex model could theoretically produce small variations in bounding-box position that have little influence on mAP50 but alter the included temperature pixels. This interpretation remains hypothetical and should be tested using direct analyses of bounding-box displacement, area, and overlap with the manually defined auricular region.

YOLO11n was ultimately selected because it showed relatively stable performance across both mean- and maximum-temperature tasks while maintaining a high inference speed of 164 FPS. It therefore provided a favorable balance among detection performance, temperature-extraction agreement, and computational efficiency. However, the selected model should not be interpreted as universally superior to the other architectures because its ranking depended partly on the weighting scheme used in the multi-criteria analysis.

### 4.3. Methodological Limitations

Several methodological limitations should be considered when interpreting the findings.

First, both manual annotation and YOLO-based detection used rectangular bounding boxes. Although this approach ensured that automated and reference measurements were evaluated using geometrically comparable regions, a rectangular box cannot precisely follow the irregular outline of an elephant ear. Consequently, some boxes may have included adjacent areas of the head, torso, or thermal background. This limitation may be particularly important for maximum-temperature extraction. A small number of non-auricular pixels with unusually high temperatures could substantially alter the extracted maximum value. Pixel-level semantic or instance-segmentation methods may therefore provide more accurate delineation of the auricular region. Shu et al., for example, used an improved U-Net architecture to segment facial thermal regions in dairy cows and automatically extract regional temperatures [25]. Future studies could similarly evaluate YOLO segmentation models or other segmentation architectures for precise delineation of the elephant auricle.

Second, infrared surface-temperature measurements are influenced by ambient temperature, relative humidity, wind speed, direct solar radiation, surface moisture, camera distance, viewing angle, emissivity, and reflected background radiation [12,27]. Although image acquisition in the present study was conducted under shaded conditions and efforts were made to avoid visibly wet body surfaces, environmental effects were not systematically corrected after temperature extraction. A recent rabbit study incorporated environmental variables into a machine-learning-based infrared temperature correction model [38]. Regarding microclimate measurement, ambient temperature and relative humidity were recorded at a height of 10 cm above ground level, which captures the near-ground microclimate experienced by resting elephants but does not fully represent the air conditions at the height of the auricle. This mismatch may limit the accuracy of any subsequent environmental correction of surface temperature values. For imaging distance, all thermograms were acquired at a standardized 5 m distance, which is consistent with standard veterinary thermography protocols and ensures that the infrared spot size is sufficiently small relative to the auricular region for reliable regional temperature measurement. Nevertheless, this fixed operating distance means that the temperature extraction performance has not been validated for longer-range imaging scenarios typical of wild elephant monitoring. The emissivity setting of 0.98 was based on the value commonly applied to human skin. The suitability of this value for Asian elephant skin has not been independently validated in the present study. Species-specific calibration using contact surface-temperature measurements or a reference blackbody would strengthen the thermometric interpretation of future studies.

Third, the Bland–Altman analyses evaluated agreement between temperatures extracted automatically and manually from the same infrared temperature matrices. The small biases observed for mean temperature therefore demonstrate strong agreement between two region-selection procedures. They do not demonstrate that the infrared camera measured the true physical temperature of the elephant ear with an error below 0.15 °C. The stated accuracy of the Fluke TiS55+ camera was ±2 °C under specified operating conditions. Accordingly, algorithmic extraction agreement and absolute thermometric accuracy should be treated as separate properties. Similarly, agreement between automated and manual auricular temperatures does not establish equivalent accuracy in estimating rectal or core body temperature.

Finally, although the repeated-measures correlation analysis used to evaluate associations between auricular and rectal temperature explicitly accounted for the non-independence of repeated observations from the same individuals, no environmental covariates were statistically controlled for in the correlation models. The physiological observations were obtained from eight semi-captive elephants maintained at one study site. Although 425 temporally matched measurements were collected, individual-level variation in age, sex, body mass, vascular regulation, skin condition, behavior, and health status may still influence the relationship between auricular and rectal temperature. Future analyses could further incorporate ambient temperature, relative humidity and time of day as covariates in mixed-effects models to better isolate the core–peripheral temperature relationship independent of environmental confounding. Validation using elephants from additional institutions or populations would also be required before the observed relationships could be generalized to the species as a whole.

### 4.4. Future Development of Core-Temperature Prediction

The present study successfully developed an automated method for detecting the outer ear and extracting auricular surface temperature. However, it did not establish a sufficiently reliable model for predicting rectal or core body temperature from outer-ear temperature.

Future studies should record a more complete set of microclimatic variables, including ambient temperature, relative humidity, wind speed, solar radiation, and potentially precipitation or surface wetness. These variables could be incorporated as covariates in multiple-regression or machine-learning models.

Machine-learning approaches have previously been used to estimate internal temperature or thermal status from infrared surface measurements. Joy et al. combined regional infrared temperatures with machine-learning techniques to assess heat stress and estimate rectal temperature in sheep [28]. Yuan et al. compared multiple predictive models for estimating core temperature in female rabbits from infrared thermographic measurements [29]. Pacheco et al. used deep learning to classify thermal conditions in dairy cows based on infrared images [30]. These studies indicate that thermal prediction generally benefits from integrating surface-temperature information with environmental and physiological covariates.

For Asian elephants, future predictive models could include: maximum and mean outer-ear temperature; temperature distribution across the auricle; ambient temperature and relative humidity; wind speed and solar radiation; time of day; elephant identity; age, sex, and body mass; recent physical activity; ear-flapping behavior; and health or inflammatory status.

Potential modeling approaches include mixed-effects regression, random forests, extreme gradient boosting, and deep neural networks. Model development should include animal-level cross-validation and evaluation on independent elephants to prevent overestimation of predictive performance.

### 4.5. Potential Applications in Elephant Management and Conservation

The automated auricular temperature extraction pipeline developed in this study is directly applicable to routine management of captive and semi-captive Asian elephants. The lightweight YOLO11n architecture, with its 164 FPS inference speed, is compatible with real-time processing of thermal imagery from fixed monitoring cameras, enabling high-frequency, non-invasive monitoring of auricular surface temperature without labor-intensive manual image processing.

Repeated automated monitoring can support animal care staff in identifying deviations from individual baseline thermal patterns, which may indicate changes in environmental heat load, physical activity, or peripheral circulatory status. The edge-deployment potential of the lightweight model also supports implementation at field sites with limited computational infrastructure or internet connectivity, where local image processing is preferable. This pattern of lightweight model deployment and on-device inference aligns with the augmented intelligence of things paradigm, in which distributed task offloading from central servers to edge nodes reduces reliance on continuous network connectivity and improves real-time responsiveness in field monitoring systems. This architectural framework has been validated for secure, efficient operation in resource-constrained IoT deployment scenarios, providing methodological support for the edge implementation of our thermal monitoring pipeline [39].

It must be emphasized that the system is not currently validated as a diagnostic tool for fever, infection, or heat stress, and clinical warning thresholds remain to be established. Extension to wild elephant monitoring via remote platforms such as unmanned aerial vehicles represents a longer-term research direction, but would require substantial additional validation given challenges of variable imaging distance, vegetation obstruction, and unconstrained animal posture.

Overall, the primary immediate value of the system lies in standardized, automated extraction of auricular surface temperature from infrared thermograms, providing a methodological foundation for future thermal physiology studies and welfare monitoring applications in managed Asian elephants.

## 5. Conclusions

This study develops and validates a non-invasive deep-learning framework for automated outer-ear detection and auricular surface temperature extraction from Asian elephant infrared thermograms, with a core novelty of integrating prior physiological validation of thermal target regions into automated detection pipeline development.

Among the three body regions examined, the outer ear showed the strongest positive association with rectal temperature, supporting its role as a physiologically informative region for non-invasive thermal monitoring. Nevertheless, the moderate strength of this relationship means auricular temperature cannot directly substitute for core body temperature and should be interpreted as a peripheral thermal indicator.

All eight lightweight YOLO models demonstrated strong outer-ear detection performance. A key methodological finding is that conventional object-detection metrics alone are insufficient for selecting models for thermographic measurement applications, because minor differences in bounding-box placement can substantially affect extracted thermal values, particularly maximum temperature. YOLO11n provided the most balanced overall performance across detection accuracy, temperature-extraction agreement, and inference speed, and was selected as the preferred model for practical deployment.

The proposed framework offers a rapid, non-invasive, and computationally efficient method for standardized auricular surface temperature monitoring in managed Asian elephants. Its immediate application lies in automated extraction of peripheral thermal measurements, rather than direct core temperature prediction or clinical diagnosis of fever and heat stress. Further validation across expanded populations and environmental conditions will support extension to broader use cases.

## Figures and Tables

**Table 1:** The indicators included in the evaluation and their weights.

Indicator	Weight
Detection rate	0.2
Absolute bias	0.15
Standard-deviation ratio	0.15
Correlation coefficient	0.15
Centered RMSD	0.15
F1 score	0.1
mAP50	0.1

**Table 2:** Correlations between Asian elephant rectal temperature and environmental and regional surface-temperature variables.

	*r_rm_*	dof	*p*	95% CI
AT	0.305	417	<0.001	[0.22, 0.39]
RH	−0.241	417	<0.001	[−0.33, −0.15]
HT_avg_	0.362	417	<0.001	[0.27, 0.45]
ET_avg_	0.395	417	<0.001	[0.3, 0.48]
T&LT_avg_	0.331	417	<0.001	[0.24, 0.42]
HT_max_	0.392	417	<0.001	[0.3, 0.48]
ET_max_	0.307	417	<0.001	[0.21, 0.4]
T&LT_max_	0.381	417	<0.001	[0.29, 0.47]

AT, ambient temperature; RH, relative humidity; HTavg, mean head temperature; ETavg, mean outer-ear temperature; T&LTavg, mean torso-and-limb temperature; HTmax, maximum head temperature; ETmax, maximum outer-ear temperature; T&LTmax, maximum torso-and-limb temperature.

**Table 3:** Intrinsic detection performance of the eight YOLO models.

Model	Training Images	Validation Images	Precision (%)	Recall (%)	F1 (%)	mAP50	FPS
YOLOv5n	1974	204	85.5	89.8	87.6	0.923	213
YOLOv5s			90.2	90.4	90.3	0.92	108
YOLOv8n			91.9	91.8	91.8	0.936	133
YOLOv8s			92.9	92.4	92.6	0.94	99
YOLO11n			91.8	90.9	91.3	0.933	164
YOLO11s			90.0	91.3	90.6	0.929	110
YOLO26n			90.8	90.3	90.5	0.936	189
YOLO26s			89.8	91.4	90.6	0.947	53

**Table 4:** Weighted rankings of the eight YOLO models for mean- and maximum-temperature extraction.

Rank	Mean-Temperature Model	Composite Score	Maximum-Temperature Model	Composite Score
1	YOLO11n	0.727	YOLOv8n	0.861
2	YOLOv8n	0.628	YOLO26n	0.815
3	YOLO26s	0.445	YOLO11n	0.776
4	YOLOv8s	0.431	YOLOv8s	0.770
5	YOLO26n	0.428	YOLO11s	0.663
6	YOLO11s	0.400	YOLO26s	0.633
7	YOLOv5n	0.278	YOLOv5n	0.556
8	YOLOv5s	0.196	YOLOv5s	0.157

## Data Availability

The original contributions presented in this study are included in the article/Supplementary Material. Further inquiries can be directed to the corresponding author.

## Supplementary Materials

The following supporting information can be downloaded at: https://www.mdpi.com/article/10.3390/ani16182870/s1, Table S1: Information of 8 Asian elephants for rectal temperature measurement; Table S2: Detailed results of the Bland–Altman analysis (mean temperature and max temperature); Table S3: Detailed results of the Taylor-diagram (mean temperature and max temperature) and Table S4: Sensitivity analysis results for weight assignments. In addition, the dataset for statistical analysis and the operation process of Python are presented separately.

## Abbreviations

The following abbreviations are used in this manuscript:

AT	Ambient temperature
ET	Outer-ear temperature
F1	Harmonic mean of precision and recall
FN	False-negative detections
FP	False-positive detections
FPS	Frames per second
HT	Head temperature
IoU	Intersection over union
IRT	Infrared thermography
LoA	Limits of agreement
mAP50	Mean Average Precision at an IoU threshold of 0.50
P_r_	Precision
R_c_	Recall
RH	Relative humidity
RMSD	Centered root-mean-square difference
RT	Rectal temperature
T&LT	Torso-and-limb temperature
TP	True-positive detections
YOLO	You Only Look Once
