# Supplementary material for: Automated Auricular Surface Temperature Monitoring in Asian Elephants Using Deep Learning and Infrared Thermography
Source: Animals (Basel). 2026 Sep 11;16(18):2870. doi: 10.3390/ani16182870 (PMC13603298; doi:10.3390/ani16182870)
Supplement: Supplementary file 1 [file animals-16-02870-s001.zip › animals-4535226-supplementary.pdf]

Table S1. Information of 8 Asian elephants for rectal temperature measurement.

Individual ID	Sex	Age(year)
ZZ	Male	3
JJ	Female	6
JX	Male	8
YN	Female	11
XQ	Male	12
RR	Female	24
YL	Female	Around 30
PP	Female	Around 40

Table S2. Detailed results of the Bland-Altman analysis(mean temperature and max temperature)

Model	Detection Rate(%)	Bias_mean (° C)	LoA_mean (° C)	Bias_max (° C)	LoA_max (° C)
YOLOv5n	91.5	-0.02	(-0.77 , 0.72)	0.27	(-5.54 , 6.07)
YOLOv5s	88.3	-0.10	(-0.71 , 0.50)	-0.03	(-2.11 , 2.05)
YOLOv8n	94.7	-0.15	(-1.30 , 1.00)	-0.29	(-2.44 , 1.85)
YOLOv8s	89.4	-0.12	(-0.86 , 0.62)	-0.12	(-1.14 , 0.89)
YOLO11n	93.6	-0.09	(-0.92 , 0.73)	-0.18	(-1.56 , 1.21)
YOLO11s	91.5	-0.09	(-0.72 , 0.55)	0.27	(-6.19 , 6.74)
YOLO26n	92.6	-0.04	(-0.69 , 0.62)	-0.06	(-1.50 , 1.39)
YOLO26s	85.1	-0.08	(-0.64 , 0.49)	-0.23	(-2.16 , 1.70)

Table S3. Detailed results of the Taylor-diagram(mean temperature and max temperature)

Model	σ_mean	r_mean	RMSD_ mean	σ_max	r_max	RMSD_ max
Reference	4.663	1	0	3.084	1	0
YOLOv5n	4.780	0.998	0.328	3.157	0.979	0.639
YOLOv5s	4.770	0.998	0.312	3.328	0.942	1.115
YOLOv8n	4.782	0.998	0.317	3.112	0.972	0.758
YOLOv8s	4.790	0.998	0.312	3.111	0.984	0.565
YOLO11n	4.729	0.998	0.322	3.112	0.979	0.662
YOLO11s	4.777	0.999	0.262	3.133	0.983	0.593
YOLO26n	4.770	0.999	0.282	3.113	0.982	0.600
YOLO26s	4.766	0.998	0.309	3.122	0.979	0.656

Table S4. Sensitivity analysis results for weight assignments

Model	Mean temperature			Max temperature		
	Probability of ranking 1st (%)	Probability of ranking top 3 (%)	Average rank	Probability of ranking 1st (%)	Probability of ranking top 3 (%)	Average rank
YOLOv5n	0	0	7	0	0	7
YOLOv5s	0	0	8	0	0	8
YOLOv8n	0	100	2	100	100	1
YOLOv8s	0	12.1	4.26	0	35.4	3.65
YOLO11n	100	100	1	0	64.6	3.35
YOLO11s	0	0	5.98	0	0	5.11
YOLO26n	0	21.7	4.25	0	100	2
YOLO26s	0	66.2	3.51	0	0	5.89
